# The Safety and Feasibility of Single-Stage Versus Staged Laparoscopic Approach for Acute Appendicitis with Inguinal Hernia in Pediatric Patients: A Comparative Study

**DOI:** 10.3390/jcm14124243

**Published:** 2025-06-14

**Authors:** Zenon Pogorelić, Anders Ødeverp, Miro Jukić

**Affiliations:** 1 Department of Surgery, School of Medicine, University of Split, 21000 Split, Croatia; 2 Department of Pediatric Surgery, University Hospital of Split, 21000 Split, Croatia

**Keywords:** acute appendicitis, inguinal hernia, simultaneous treatment, laparoscopy, children

## Abstract

**Background:** The simultaneous surgical treatment of acute appendicitis and inguinal hernia in children is still controversial. However, there are no established guidelines for the simultaneous surgical treatment of pediatric patients with acute appendicitis and inguinal hernia. The aim of this study is to evaluate the safety and efficacy of a simultaneous laparoscopic approach for acute appendicitis and inguinal hernia in a pediatric population. **Methods:** The case records of 2254 pediatric patients who underwent appendectomy at our institution between 1 January 2012 and 1 January 2025 were reviewed. Finally, 44 patients who met the inclusion criteria and had an inguinal hernia at the time of laparoscopic appendectomy were selected for further analysis. The patients who underwent single-stage surgery (simultaneous laparoscopic appendectomy and hernia repair) were assigned to group I (*n* = 25), while the patients who underwent delayed laparoscopic hernia repair were assigned to group II (*n* = 19). The groups were compared for final outcome, complications, rate of readmissions within 30 days of index surgery, duration of surgery, and length of hospital stay. **Results:** The mean age of all the included patients was 11.5 ± 4.0 years, with males slightly outnumbering females (*n* = 25, 56.8%). The study population consisted of two comparable groups in terms of age, anthropometric measures, gender distribution, and baseline clinical characteristics. A major difference between the two methods was the operation time, which was significantly longer in the single-stage group (53.5 ± 11.2 min vs. 41.5 ± 10.9 min; *p* = 0.001). Despite the difference in operative time, the length of hospital stay (3.5 ± 2.0 days vs. 3.5 ± 2.2 days; *p* = 0.899) was almost identical between the two groups, suggesting that the additional intraoperative time was not reflected in a prolonged recovery time. In addition, postoperative complications were rare and evenly distributed between both surgical strategies (*n* = 2 (8%) vs. *n* = 2 (10.5%); *p* = 0.772). All the complications were minor and were treated conservatively. Importantly, there was no recurrence of hernia in either group during the follow-up period. **Conclusions:** From a clinical perspective, these results suggest that the single-stage approach is feasible and safe, even in complicated appendicitis, particularly in cases where the postponement of hernia repair is not desirable. The longer operative time associated with the single-stage approach must be weighed against the potential benefits of avoiding a second surgical procedure and unnecessary anesthesia, reducing overall healthcare utilization, and minimizing patient burden.

## 1. Introduction

Acute appendicitis and indirect inguinal hernias are two of the most commonly encountered surgical conditions operated in the pediatric population worldwide [1]. In general, the incidence of pediatric appendicitis is 83:100,000 and pediatric inguinal hernias is 0.8–12% [2,3,4]. Out of all of the emergency surgically treated pediatric acute abdomen disorders, acute appendicitis has a 1–8% overall rate, while the overall rate of inguinal hernias is 0.8–4.4% [5,6]. The incidence of mortality of inguinal hernias is set at 1–4% [7]. Appendicitis, however, has, over the last two decades, decreased in mortality rate reportedly due to the novel accepted gold standard diagnostic and therapeutic laparoscopic appendectomies [8]. Both conditions require timely and effective management to prevent severe complications and associated morbidity. As the inflammation of the appendix progresses from simple to complicated, perforation with a localized biological abscess formation follows with a high risk of the diffuse spreading of infection [9]. Acute appendicitis is classified as a contaminated surgical condition with a high risk for infection [10]. In comparison to appendicitis, inguinal hernias are clean, non-contaminated surgical conditions [11]. The risk of strangulation and incarceration are the main reasons why laparoscopic procedures are performed early in inguinal hernia repair in children [12,13,14]. In the modern world of surgery, less invasive laparoscopic techniques are performed to achieve satisfactory clinical results in both conditions [15,16].

After laparoscopic appendectomy was recognized as the gold standard treatment for uncomplicated appendectomy in children, the incidental detection of unrelated conditions such as inguinal hernias in this population group has increased [17,18,19]. A study of patients undergoing laparoscopic procedures showed that 13% with previously unidentified inguinal hernias were discovered [18]. Laparoscope as a diagnostic and therapeutic tool has excellent visualization of intraperitoneal organs and is, therefore, contributing to the increased inguinal hernia detection rather than an increased incidence of the disease itself [19]. The anticipated probability of concurrent inguinal hernias and appendicitis has been calculated to be 9:1,000,000; however, this number is expected to change due to the great visualization in laparoscopic procedures [20]. Furthermore, a multicentric study shoved the actual frequency of them occurring together to be 5.7% in laparoscopic appendectomies performed in children [21]. The laparoscopic procedure is, therefore, efficient in diagnosing disorders simultaneously in addition to its immense potential to combine two or more treatment options in the same surgical procedure for concurrent conditions [22,23]. For example, great results have been found in conjoint laparoscopic appendectomies and cholecystectomies [24]. However, since both of these organs are primarily potentially contaminated, there is no clear violation of the principles of aseptic surgery when combining these two surgical interventions laparoscopically [24]. In comparison, since inguinal hernia repair is a clean surgical disease, its intervention has been avoided when contaminated surgical disease interventions have been performed [25,26]. Even though appendicitis and inguinal hernias are both treated laparoscopically, their management has differed largely based on the principles of contamination, the risk for infection, and therefore, hernia recurrence [25,27,28,29,30,31]. Recently, reports have been made regarding a two-in-one, single-stage laparoscopic treatment potential for concurrent acute appendicitis and inguinal hernia in a pediatric population. One special clinical entity worth noting is the Amyand hernia, which refers to the presence of the appendix within an inguinal hernia sac, first described by Claudius Amyand in the 18th century. While rare in the pediatric population, its presence may complicate both the diagnosis and management of acute appendicitis and inguinal hernias. Amyand hernias blur the lines between contaminated and clean surgical fields, particularly when the appendix is inflamed or perforated. The approach for these cases often parallels decisions made in the context of concurrent appendicitis and inguinal hernia, underscoring the relevance of exploring single-stage versus staged management strategies [10].

The first of the largest series of retrospective comparative trials between single-stage laparoscopic treatments versus two-stage laparoscopic procedures in this population group was documented by Li et al. [21]. This multicentric study revealed significantly shorter hospital stays, greater cost-effectiveness, and less psychological and surgery-related stress on the children in the simultaneous laparoscopically treated group [21]. The same group demonstrated maximum benefits through safety, effectiveness, and feasibility. Few reports exist to date due to the long-lasting idea of the contaminated nature of appendectomy and the associated high risk for surgical site infections and recurrence of inguinal hernias due to wound contamination [21]. Dayi et al. conducted a single-center study on the topic of simultaneous laparoscopic appendectomy and inguinal hernia repair and showed that this is possible with almost identical results to Li et al. [19]. At the same time, there was no increase in complication rate because of the strictly adopted inclusion criteria in both reports. They excluded patients with severe abdominal infections such as perforated appendicitis, gangrenous appendicitis, and peritonitis [19,21]. Furthermore, Dayi et al. emphasized that the risk of inguinal hernia recurrence in girls can be almost eliminated through the sutureless Burnia technique [19,32,33].

There is no generally accepted consensus or guidelines on treating the pediatric population with concomitant appendicitis and inguinal hernia [19]. We conducted a single-center retrospective comparative study between single-stage and two-stage laparoscopic intervention of concurrent acute appendicitis and inguinal hernia in the pediatric population. The treatment approach for concurrent acute appendicitis and inguinal hernia has been reported in recent years but there is still a lack of enough evidence in the literature to be able to develop a generalized treatment protocol [21]. We evaluated the safety and feasibility of the single-stage procedure. In the age of laparoscopic surgery, the perspective on the traditional treatment protocols is postulated to change. Our study aims to contribute to the pool of evidence so that a novel treatment protocol for concurrent acute appendicitis and inguinal hernia can be developed in the future. We hypothesized that the single-stage approach can be a practical alternative to the traditional two-stage approach, particularly in cases where inguinal hernia repair should not be postponed, including cases of simple and complicated appendicitis with inguinal hernia.

## 2. Methods

### 2.1. Patients

The case files of 2254 pediatric patients who underwent appendectomy in the Department of Pediatric Surgery at the University Hospital of Split between 1 January 2012 and 1 January 2025 were evaluated. The inclusion criteria were pediatric patients aged 0 to 17 years who underwent laparoscopic appendectomy and had an inguinal hernia at the time of surgery, followed up for a minimum period of 3 months. Patients who had undergone open appendectomy, patients who did not have an inguinal hernia at the time of surgery, and patients with incomplete data in the medical records were excluded from the study. Of the total of 2254 patients who underwent appendectomy, 878 patients were initially excluded because they had undergone open appendectomy. Of the remaining 1376 patients, 11 were excluded because of incomplete data, 5 because of a follow-up period of less than three months, and 1316 because they had no concomitant hernia at the time of laparoscopic appendectomy. Finally, 44 patients who had an inguinal hernia at the time of laparoscopic appendectomy were selected for further investigation.

**Figure 1:**
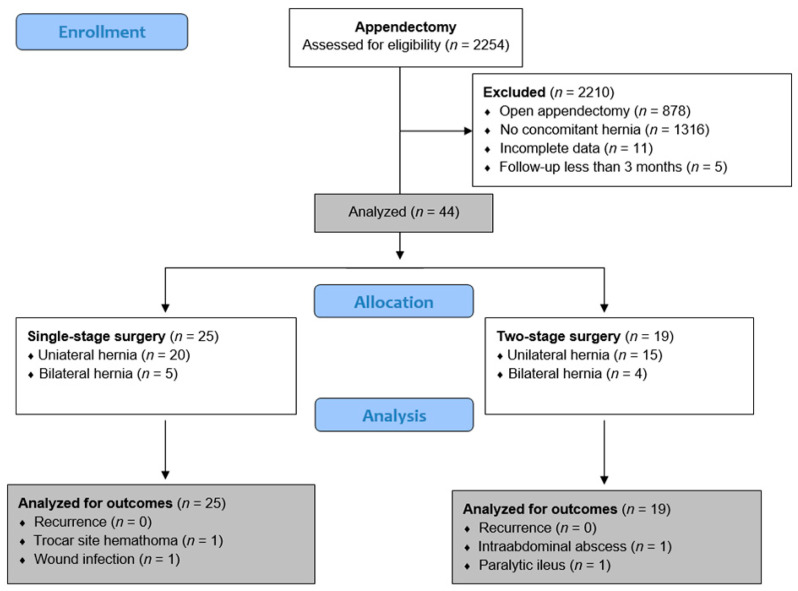
Flow chart of the study.

For the purposes of this study, these patients were divided into two study groups based on the timing of hernia repair. The patients who underwent single-stage surgery (simultaneous laparoscopic appendectomy and hernia repair) were assigned to group I (*n* = 25), while the patients who underwent delayed laparoscopic hernia repair were assigned to group II (*n* = 19). Moreover, the patients were subdivided into subgroups depending on appendicitis severity (simple or complicated appendicitis). The decision to perform single-stage or two-stage surgery was based on the surgeon’s clinical judgment. The flow chart of the study is shown in Figure 1.

### 2.2. Ethical Aspects

The study was conducted in accordance with the ethical principles outlined in the Declaration of Helsinki and its subsequent revisions or equivalent ethical standards. Ethical approval was granted by the Institutional Review Board of the University Hospital of Split (approval number: 500-03/25-01/75; date of approval: 2 April 2025).

### 2.3. Outcomes of the Study and Definitions

The primary outcome measure was the success of the hernia operation, specifically defined by the absence of hernia recurrence during the follow-up period. The secondary outcome measures were the occurrence of early or late complications, the rate of readmissions within 30 days after the index operation (ReAd) [34], the duration of the operation, and the length of hospital stay.

In this study, feasibility was defined as the ability to successfully complete both appendectomy and hernia repair laparoscopically in the same surgical session without conversion to open surgery. Safety encompassed both intraoperative and postoperative outcomes, including intraoperative complications, postoperative infections, 30-day readmission, and recurrence of inguinal hernia during follow-up.

### 2.4. Study Design

All the patients enrolled in the study underwent standard laparoscopic appendectomy for acute appendicitis. The diagnosis of acute appendicitis was based on a combination of laboratory findings (inflammatory markers, white blood cells, neutrophil granulocytes, and C-reactive protein), preoperative imaging (abdominal ultrasonography), AIR score, and clinical examination [35]. The final decision was made on the basis of the surgeon’s opinion. Intraoperatively, the appendix was classified as simple (cathartic, phlegmonous, or gangrenous) or complicated (inflammation of the peritoneum, pus in the abdominal cavity, visible hole in the appendix, or free fecolith in the abdominal cavity). The hernia was diagnosed preoperatively (in symptomatic cases) or intraoperatively if an open internal ring of the inguinal canal was found during the laparoscopic exploration of the abdominal cavity. The following data were collected for each patient included in the study: age, gender, height, body weight, body mass index, comorbidities, ASA classification, lateralization of the hernia, type of appendicitis, early and late complications, hernia recurrence, reoperation rate, ReAd, duration of surgery, duration of hospital stay, and duration of follow-up.

### 2.5. Surgical Technique

General anesthesia with an endotracheal tube was performed on all the patients. The patient was placed in the supine position. A 5 mm incision was made supraumbilically. Depending on the patient’s weight and age, a pneumoperitoneum of 8–12 mmHg was achieved. As soon as the pneumoperitoneum was reached, a 5 mm trocar for the camera was inserted through the supraumbilical incision. After exploring the abdominal cavity, two additional trocars were placed in position for laparoscopic appendectomy (5 mm trocar under the right costal arch in the midclavicular line and 10 mm trocar in the left iliac fossa). After the exploration of the abdominal cavity, the laparoscopic appendectomy was performed using the standard technique described previously [36].

In the single-stage group, a percutaneous internal ring suturing (PIRS) was performed after the appendectomy using the standard technique described previously [37]. In the two-stage group, hernia repair was performed after three to six months.

The choice between single-stage and two-stage surgery was based on the operating surgeon’s clinical judgment, taking into account the severity of appendicitis, the presence of peritoneal contamination, the patient’s overall clinical status, and anesthetic risk.

### 2.6. Postoperative Protocol and Follow-Up

Following surgery, the patients were maintained on intravenous fluids until the initiation of oral intake, which was commenced a few hours postoperatively at the discretion of the attending surgeon. Analgesia was provided using either paracetamol (Paracetamol Kabi, Fresenius Kabi, Zagreb, Croatia) at a dose of 15 mg/kg or diclofenac (Lek Pharmaceuticals, Ljubljana, Slovenia) at 1 mg/kg. Antibiotic therapy was generally not required for the patients diagnosed with simple appendicitis. In contrast, all the patients with complicated acute appendicitis received antimicrobial treatment, most commonly a combination of gentamicin (Gentamicin, Belupo, Koprivnica, Croatia) at 3–5 mg/kg once daily and metronidazole (Metronidazole, B. Braun Adria, Zagreb, Croatia) at 7.5 mg/kg three times daily. If this regimen proved ineffective, the antibiotic selection was adjusted according to the antibiogram results. Discharge criteria included the absence of significant pain, fever, or vomiting, and the ability to tolerate oral nutrition. Post-discharge follow-up was conducted at our outpatient clinic on postoperative days 7 and 30.

### 2.7. Statistical Analysis

Statistical analysis was performed using the Statistical Package for Social Sciences (SPSS, Version 28.0, IBM Corp., Armonk, NY, USA). The Shapiro–Wilk test was conducted to assess the normality of the data distribution. Since all the continuous variables followed a normal distribution, they were reported as mean and standard deviation (SD). The categorical variables were described using absolute numbers and percentages. Comparisons of continuous variables between the groups were performed using the independent samples *t*-test. The categorical variables were analyzed using the chi-square test, with Fisher’s exact test employed in cases of low expected frequencies. A *p*-value of less than 0.05 was considered indicative of statistical significance.

## 3. Results

The study cohort comprised a total of 44 patients, 25 of whom were assigned to the single-stage surgical group and 19 to the two-stage surgical group. The mean age of all the included patients was 11.5 ± 4.0 years, with males slightly outnumbering females (*n* = 25, 56.8%). The study population consisted of two comparable groups in terms of age, anthropometric measures, gender distribution, and baseline clinical characteristics. Both the single-stage and two-stage groups showed no significant differences in age (*p* = 0.970), height (*p* = 0.478), weight (*p* = 0.221), or BMI (*p* = 0.567), indicating a well-matched cohort.

The distribution of male and female patients was also balanced (*p* = 0.625), and the severity of appendicitis categorized as simple or complicated was almost identical in both groups (*p* = 0.954). The presence of unilateral or bilateral hernias (*p* = 0.931), the proportion of symptomatic hernias (*p* = 0.878), and the frequency of comorbidities (*p* = 0.772) were also similar across both approaches. Moreover, the ASA classification, which provides an assessment of preoperative risk, did not differ significantly between the groups (*p* = 0.984). The patient characteristics and demographic data are presented in Table 1. These similarities in baseline characteristics suggest that any observed differences in surgical outcomes can be attributed primarily to the choice of surgical technique rather than patient-related factors.

A major difference between the two methods was the operation time, which was significantly longer in the single-stage group (53.5 ± 11.2 min vs. 41.5 ± 10.9 min; *p* = 0.001). This difference was observed both in the entire cohort and in the subgroups of patients with simple and complicated appendicitis. The longer duration of surgery in the single-stage group is to be expected given the concomitant nature of the procedures (appendectomy and hernia repair were performed in the single-stage group, whereas only appendectomy was performed in the index surgery in the second group). Despite the difference in operative time, the length of hospital stay (3.5 ± 2.0 days vs. 3.5 ± 2.2 days; *p* = 0.899) was almost identical between the two groups, suggesting that the additional intraoperative time was not reflected in a prolonged recovery time. In addition, postoperative complications were rare and evenly distributed between both surgical strategies (*n* = 2 (8%) vs. *n* = 2 (10.5%); *p* = 0.772). Importantly, there was no recurrence of hernia in either group during the follow-up period. This finding suggests that performing hernia repair concurrently with appendectomy does not compromise long-term surgical outcomes, further supporting the feasibility of the single-stage approach. The treatment outcomes of the patients are shown in Table 2.

The analysis of postoperative complications did not reveal any major problems in favor of one or the other approach. Minor complications such as wound infection, hematoma, intra-abdominal abscess and paralytic ileus were observed in both groups, but their incidence remained low and no cases of anastomotic leakage or diffuse peritonitis were reported. The absence of hernia recurrence is further evidence of the longevity of single-stage repair. All complications were treated conservatively without the need for surgical intervention. The postoperative complications and their distribution among the individual groups are shown in Table 3.

## 4. Discussion

Our comparative single-centered study of the surgical treatment approach for the pediatric population with concurrent acute appendicitis and inguinal hernias compared a single-stage and a staged surgically treated group. As expected, the single-stage procedure resulted in longer operation time; however, as the hospital time was similar in both patient groups, it is instead a clear indicator of faster recovery time favoring the single-stage procedure, especially since the rates of postoperative complications were rarely seen in both groups, and therefore, did not contribute significantly to the recovery or long-term outcomes of either group. Both approaches culminated in similar and excellent safety profiles with few wound infection complications and no instances of life-threatening cases of diffuse peritonitis. Therefore, the single-stage approach, treating primarily contaminated and uncontaminated together, is of similar safety margin as a two-stage procedure. Furthermore, no hernia recurrences were observed, reinforcing that a single-stage approach is effective and durable in its nature. It is evident that the feasibility of the single-stage laparoscopic appendectomy and hernia repair risk through longer operation time is balanced out with no subsequent need for repeat hospitalization or anesthesia. The single-stage approach showed statistically similar safety, effectiveness, durability, and feasibility without compromising the patient’s long-term outcomes, surgical site infections, or recurrences of inguinal hernias, which have been previously reported concerns in the literature with the single-stage approach [21].

To date, only a few reports discussing the differences between single-stage and two-stage treatment approaches for the pediatric population with concurrent acute appendicitis and inguinal hernia are available. However, according to the literature, there has been an increased interest in recent years on this topic as it has been emphasized that a new treatment approach guideline for this population group is under demand [19,21,38,39,40]. The great outcomes of the single-stage group in our study largely reflect the surgical techniques used, the suture-less harmonic scalpel laparoscopic appendectomy and PIRS. Clipless appendectomy is associated with fewer complications and shorter surgical duration compared to standard appendectomy techniques [36]. Furthermore, PIRS has emerged as a safe and effective method of treating inguinal hernias in young children, also with concurrent appendicitis [37,39,40]. PIRS has recently been documented as a safe procedure in multiple studies on acute infectious disorders and appendectomies concurrent with inguinal hernia repair [38,39,40]. Prior to the implementation of this method in the single-stage approach, there has been an associated worrisome risk for inguinal hernia recurrence, especially in girls, where a secondary intervention through the Burnia technique has been discussed and used for its great potential in girls [19,21,32,33,41]. Importantly, our study showed no inguinal recurrences with PIRS in either sex and no need for repeat secondary interventions through the Burnia technique. Our single-stage treatment approach appears, therefore, safer, more feasible, more durable, and more effective in both genders, also suggested by multiple other studies [38,39,40], in comparison to the first documented single-stage approaches used in this pediatric population group [19,21]. As the treatment outcomes in the single-stage groups of previously reported studies performed by Li et. Al. and Dayi et al. were largely due to the strict monitoring of the inclusion and exclusion criteria, we followed the same principles but with a modification [19,21]. In addition to treating simple appendicitis and concurrent inguinal hernia, we also treated complicated appendicitis with concurrent inguinal hernia. Complicated appendicitis is known for its high risk of infection and inguinal recurrence and has, therefore, been excluded in previously reported studies [19,21].

We show that in addition to simple appendectomies, complicated appendectomies can be treated in a single-stage harmonic scalpel laparoscopic appendectomy and PIRS intervention in a pediatric population with no statistical difference to hernia reoccurrence. This suggests a viable treatment strategy for managing both contaminated and clean surgical conditions concurrently with a clear opposing statement to the traditional concerns of operating them in a single procedure.

The postoperative complications were minor and few without statistically significant differences between the two groups. Importantly, similar results were reported by the multicentric study of Li et al. which had a much larger study population number [21]. This similarity might serve to generalize our findings more since a clear limitation of our study is the low number of patients involved. Furthermore, the single-center study experience is another limitation, but when compared with the multicentric study, our study shows almost identical results. Every center differs in its guidelines and approach for patients. Since our findings are a single-center experience, our findings have limited generalizing potential alone. Instead, our results serve to expand and add to the evolving evidence pool in the treatment approach for the pediatric population with concurrent appendicitis and inguinal hernia. According to the other single-center studies that exist in the literature to date, our experience shows, yet again, almost identical results [19,39,40]. Another limitation is the retrospective nature of the study itself as the initial intent was not based on the sole purpose of the creation of this study. Retrospective studies have a well-known lack of control of variables and risk of inconsistencies as the existing data may be inaccurate in comparison to prospective studies [42]. We needed to exclude a few patients based on the lack of data which could have potentially contributed significantly to the outcome of the results. Even though the operation was the surgeon’s decision, there is an unwanted selection bias due to the non-randomized nature of the study itself. In addition, the allocation of patients to either single-stage or two-stage treatment groups was based on the surgeon’s discretion, which inherently introduces a risk of selection bias. For example, surgeons may have preferentially selected less complicated cases for the single-stage approach or may have avoided simultaneous procedures in patients perceived as higher risk. This non-random allocation could have influenced outcomes such as complication rates, operative time, or length of hospital stay.

In the future, it would be of great interest to create prospective randomized control trials with the sole purpose of comparing single-stage and two-staged laparoscopic appendectomy and inguinal hernia repair in a pediatric population. Carrying out more multicentric studies would diversify the results with a larger study population and multiple different centers involved. This would increase the applicability of the results to the general population. The guidelines and policies regarding the treatment approach for this specific population group are under great demand and in high need of more studies to be performed. The evidence pool in favor of the single-stage procedure is increasing in size and shows great results. Furthermore, more studies need to be performed on complicated appendicitis cases with inguinal hernias as our study is the first to implement them in a comparative study and clearly shows a great treatment outcome in our single-center experience. A prospective comparative study on the treatment outcomes between simple and complicated appendicitis with inguinal hernia repair in a single-stage treatment approach could be the next step. It would be of great interest to compare them directly as our findings clearly indicate no statistically significant difference between the single-stage and two-stage approach on these patients. The potential guideline for the treatment approach can, therefore, in the future, implement not only simple appendicitis cases but also contaminated appendicitis cases with concurrent inguinal hernia.

## 5. Conclusions

A single-stage treatment approach for pediatric patients with acute appendicitis and inguinal hernia is feasible and safe. As the time of hospitalization did not differ between the single-stage and staged procedure groups, even with an increased operative time, it is an effective intervention. The PIRS method appears durable as it did not show any inguinal hernia recurrences when exposed to simple appendicitis or the more contaminated, complicated acute appendicitis. As there is also no need for repeat hospitalization or anesthesia, it reduces overall healthcare utilization, which is a clear benefit. Importantly, the long-term outcomes and benefits are in favor of the single-stage treatment approach for the pediatric population with concurrent acute appendicitis and inguinal hernia. However, the associated prolonged operation time needs to be weighed against these benefits before deciding the treatment approach.

## Figures and Tables

**Table 1:** Patient characteristics and demographic data.

Variables	Single-Stage Group (*n* = 25)	Two-Stage Group (*n* = 19)	*p*
Age, years [mean ± SD]	11.5 ± 3.9	11.5 ± 4.2	0.970 *
Height, cm [mean ± SD]	155.7 ± 21.9	155.4 ± 20.8	0.478 *
Weight, kg [mean ± SD]	44.4 ± 17.5	48.7 ± 19.2	0.221 *
BMI, kg/m^2^ [mean ± SD]	18.9 ± 2.1	19.3 ± 2.4	0.567 *
Gender [*n* (%)]			
Male	15 (60)	10 (52.6)	0.625 ^‡^
Female	10 (40)	9 (47.4)	
Type of appendicitis [*n* (%)]			
Simple	16 (64)	12 (63.2)	0.954 ^‡^
Complicated	9 (36)	7 (36.8)	
Type of hernia [*n* (%)]			
Unilateral	20 (80)	15 (79)	0.931 ^†^
Bilateral	5 (20)	4 (21)	
Symptomatic hernia [*n* (%)]	3 (12)	2 (10.5)	0.878 ^†^
Comorbidities [*n* (%)]	2 (8)	2 (10.5)	0.772 ^†^
ASA classification [n (%)]			
ASA I	21 (86)	16 (84.2)	0.984 ^†^
ASA II	4 (16)	3 (15.8)	

BMI—body mass index; SD—standard deviation, ASA—American Society of Anesthesiologists. * independent *t*-test; ^‡^ chi-square test; ^†^ Fisher’s exact text.

**Table 2:** Treatment outcomes of the patients.

Variables	Single-Stage Group (*n* = 25)	Two-Stage Group (*n* = 19)	*p*
Entire cohort			
Operative time, min [mean ± SD]	53.5 ± 11.2	41.5 ± 10.9	0.001 *
LOS, days [mean ± SD]	3.5 ± 2.0	3.5 ± 2.2	0.899 *
Postoperative complications [*n*, (%)]	2 (8)	2 (10.5)	0.772 ^†^
Hernia recurrence [*n*, (%)]	0 (0)	0 (0)	-
Follow-up, months [mean ± SD]	98 ± 24.1	96 ± 22.4	0.861 *
Simple appendicitis			
Operative time, min [mean ± SD]	34.7 ± 11.4	26.7 ± 12.1	0.001 *
LOS, days [mean ± SD]	2.1 ± 1.4	2.2 ± 1.7	0.919 *
Postoperative complications [*n*, (%)]	1 (4)	0 (0)	>0.999 ^†^
Hernia recurrence [*n*, (%)]	0 (0)	0 (0)	-
Follow-up, months [mean ± SD]	97 ± 22.9	85 ± 25.2	0.894 *
Complicated appendicitis			
Operative time, min [mean ± SD]	49.5 ± 13.5	38.5 ± 9.2	0.002 *
LOS, days [mean ± SD]	4.8 ± 1.9	5.0 ± 2.2	0.618 *
Postoperative complications [*n*, (%)]	1 (4)	2 (10.5)	0.569 ^†^
Hernia recurrence [*n*, (%)]	0 (0)	0 (0)	-
Follow-up, months [mean ± SD]	100 ± 23.8	98 ± 24.4	0.799 *

LOS—length of hospital stay; SD—standard deviation. * independent *t*-test; ^†^ Fisher’s exact text.

**Table 3:** Postoperative complications.

Variables	Single-Stage Group (*n* = 25)	Two-Stage Group (*n* = 19)
Anastomotic leak	0	0
Diffuse peritonitis	0	0
Intra-abdominal abscess	0	1
Paralytic ileus	0	1
Wound infection	1	0
Trocar site hemathoma	1	0
Recurrent inguinal hernia	0	0
Total	2	2

## Data Availability

The data assessed and reported here can be obtained from the authors upon reasonable request and following ethical and privacy principles.
